# ﻿A new species of *Umbilia* Jousseaume, 1884 (Mollusca, Cypraeidae) from the Pliocene fauna of the Roe Plains, Western Australia

**DOI:** 10.3897/zookeys.1169.106338

**Published:** 2023-07-05

**Authors:** Paul C. Southgate, Thane A. Militz

**Affiliations:** 1 School of Science, Technology & Engineering and Australian Centre for Pacific Islands Research, University of the Sunshine Coast, Maroochydore, Queensland 4556, Australia University of the Sunshine Coast Sippy Downs Australia

**Keywords:** Cowrie, cowry, fossil, Roe Calcarenite, taxonomy, *Umbiliatomdarraghi* sp. nov.

## Abstract

A new morphologically distinct species of cowry (family Cypraeidae Rafinesque, 1815) is described from the Late Pliocene Roe Calcarenite of the Roe Plains, Western Australia. Previously assigned to *Umbiliahesitata* (Iredale, 1916), the new species differs morphometrically from related taxa and is differentiated from *U.hesitata* by a number of shell features including a prominent, projecting protoconch, less extended posterior and anterior terminals, coarser columellar teeth extending onto the base, and well-developed, thickened anterior flanges, supporting a rounded anterior extremity with blunt anterior tips. *Umbiliatomdarraghi* sp. nov. is the third *Umbilia* species to be described from the Pliocene.

## ﻿Introduction

The Roe Calcarenite is a fossil-rich Late Pliocene deposit covering an area of around 12,000 km^2^ of the Roe Plains in south-eastern Western Australia (Kendrick et al. 1991, 1997; James et al. 2006). Fossils of a broad range of molluscs are common within the Roe Calcarenite with around two-thirds of these originally considered to represent living species (Ludbrook 1978). Six species of cowries (Gastropoda, Cypraeidae) have been reported from the Roe Calcarenite, of which five are extinct; *Austrocypraeaamae* Fehse & Kendrick, 2000, *Notocypraeadarraghi* Fehse, 2010, *N.goudeyi* Fehse, 2011, *Zoilacampestris* Darragh, 2011, and *Umbiliafodinata* (Darragh, 2011). Darragh (2002) assigned the sixth species to *U.hesitata* (Iredale, 1916), while noting that the Roe Plains is considerably further west than the western-most range of living *U.hesitata*, represented by the subspecies *U.h.suprastrata* Govaert et al., 2015. Subsequent authors have expressed uncertainty regarding assignment of these Roe Plains fossils to *U.hesitata*. Wilson and Clarkson (2004), for example, commented that the *U.hesitata*-like fossils from Roe Plains are atypical of living *U.hesitata* and noted their “rather flat base and stronger teeth”, while Lorenz (2018) referred to these fossils as an “ancestral *hesitata*-like species”. Only one living member of the genus, *Umbiliaarmeniaca* (Verco, 1912), is currently found in offshore habitats adjacent to the Roe Plains. Goudey (2015), however, noted that Roe Plains *U.hesitata*-like fossils are less inflated than the shells of living *U.armeniaca*.

Detailed study of the Roe Plains *U.hesitata*-like fossils has not previously occurred, probably because of the rarity of intact specimens. However, we recently identified a number of specimens in museum and private collections, available for study, allowing detailed morphometric examination of this taxon for the first time. Past research on fossil and extant species of *Umbilia* (Southgate et al. 2021; Southgate and Militz 2023) generated morphometric data relating to shell form (i.e., shell length, shell height, shell width, columellar and labral tooth counts, and relative mass) for each of the recognised subspecies of *U.hesitata* and *U.armeniaca*. In the present study, these data were used to support multivariate morphometric comparisons between the Roe Plains *U.hesitata*-like fossils and extant specimens of *U.hesitata* and *U.armeniaca*, at both species and subspecies levels. Results showed clear morphometric separation of the Roe Plains *U.hesitata*-like fossils from both *U.hesitata* and *U.armeniaca*, and sufficient differentiation from both living and fossil taxa to justify recognition of the Roe Plains *U.hesitata*-like fossils as a new species, described here as *Umbiliatomdarraghi* sp. nov.

## ﻿Materials and methods

### ﻿Examined materials

All examined specimens of the Roe Plains *U.hesitata*-like fossils were recovered from material excavated from the Roe Calcarenite at various sites within the general area between Madura (31°53'58"S, 127°01'11"E) and the Hampton repeater tower (31°57'52"S, 127°34'50"E), Western Australia, including the Main Roads quarry 16 km south of Madura (32°02'22"S, 127°02'50"E), which was a major source of material used in construction, upgrades, and maintenance of the Eyre Highway, which dissects the Roe Plains. Only specimens supporting accurate assessment of all morphometric characters, outlined below, were included in this study.

### ﻿Data collection

Primary data were generated for 11 specimens of the Roe Plains *U.hesitata*-like fossils. Shell length (L), shell width (W), and shell height (H) were measured to the nearest 0.1 mm using vernier callipers. Counts of columellar teeth (CT) included the posterior-most denticle that merges with the anterior edge of the columella callus bordering the posterior canal. All labral teeth (LT) were counted.

Secondary data for L, W, H, CT, and LT for extant *U.armeniaca* and *U.hesitata* were sourced from prior research. Specifically, data for the three recognised subspecies of *U.hesitata* [*U.h.hesitata* (*n* = 30), *U.h.beddomei* (*n* = 14), and *U.h.suprastrata* (*n* = 30)] were sourced from Southgate et al. (2021) and data for the four recognised subspecies of *U.armeniaca* [*U.a.armeniaca* (*n* = 51), *U.a.diprotodon* (*n* = 30), *U.a.clarksoni* (*n* = 17), and *U.a.andreyi* (*n* = 36)] were sourced from Southgate and Militz (2023). Descriptive terminology generally follows that of Lorenz (2002, 2017).

### ﻿Data analysis

Data analysis combined qualitative appraisal of key conchological features, such as the aperture, spire, columellar teeth, anterior and posterior terminals, and base, with a quantitative appraisal of overall shell form. For the quantitative component of this analysis, we adapted the multivariate approach of Southgate and Militz (2023) to compare specimens of the Roe Plains *U.hesitata*-like fossils with extant *U.armeniaca* and *U.hesitata*, after aggregating subspecies data, and to compare specimens of the Roe Plains fossils with each of the extant *U.hesitata* subspecies.

Shell form was represented by the following morphometric characters: L, width to length ratio (W/L), height to length ratio (H/L), height to width ratio (H/W), normalised LT (nLT), and normalised CT (nCT). For each specimen, nLT and nCT were calculated from LT and CT, respectively, for a hypothetical shell length of 25 mm as described by Schilder (1937) and W/L, H/L and H/W were expressed as a percentage (Lorenz 2017). While the multivariate approach of Southgate and Militz (2023) also incorporated relative mass (*sensu*Bridges and Lorenz 2013), this approach is inappropriate with fossils where the influence of mineralisation and residual matrix may compromise resulting data. Aside from this alteration, the multivariate approach taken in this study followed that of Southgate and Militz (2023). Briefly, values for the morphometrics outlined above were transformed to *Z*-scores and atypical specimens (i.e., |*Z*-score| > 3) either validated (primary data) or censored (secondary data) before computing a resemblance matrix based on Euclidean distance between specimens. Non-metric multidimensional scaling (nMDS) was then used for dimensionality reduction to permit visualisation in two dimensions. Visual observations of the nMDS configuration were validated by estimating the probability that *a priori* assigned groups (i.e., taxa) shared the same central tendency (i.e., centroid) and variation (i.e., dispersion) in shell form. Specifically, a one-factor permutational analysis of variance (PERMANOVA) was used to estimate the probability that groups shared the same central tendency in shell form; pairwise comparisons proceeded detection of a significant group effect, using PERMANOVA for each comparison and controlling for the family-wise error rate with the Holm (1979) procedure. Permutation-based tests for homogeneity of multivariate dispersions were used to compare the distance of specimens from their centroid, controlling for the family-wise error rate with the Holm (1979) procedure.

All statistical computing was performed using R (version 4.2.1) with the *stats* (R Core Team 2022) and *vegan* (Oksanen et al. 2022) packages. For statistical tests, significance was accepted at a value of *P* < 0.01 as recommended by Southgate and Militz (2023) to conservatively establish inter-group differences. Data summaries for a specific morphometric are presented in-text as means (x̄) ± standard deviation (SD) and for all morphometrics, collectively, means are presented using the “shell formula” [L (W/L-H/L-H/W) nLT: nCT] (Bridges and Lorenz 2013).

### ﻿Abbreviations

**AB** Adrian Bishop collection, Yorketown, South Australia, Australia;

**CG** Chris Goudey collection, Lara, Victoria, Australia;

**JF** Jonathan Fell collection, Melbourne, Victoria, Australia;

**MV** Museums Victoria, Melbourne, Australia;

**PH** Peter Hunt collection, Adelaide, South Australia, Australia;

**WAM**Western Australian Museum, Perth, Australia.

## ﻿Results

### ﻿Systematics

**Class Gastropoda Cuvier, 1795**

**Order Littorinimorpha Golikov & Starobogatov, 1975**

**Superfamily Cypraeoidea Rafinesque, 1815**

#### ﻿Family Cypraeidae Rafinesque, 1815

##### ﻿Genus Umbilia

Taxon classificationAnimaliaLittorinimorphaCypraeidae

Cossmann, 1903

25A9D262-0683-57CD-BF80-24CD4BF0A61A

###### Type species.

*Cypraeaumbilicata* G.B. Sowerby I, 1825 (by original designation); *Umbiliahesitata* Iredale (1916) by subsequent designation.

##### ﻿ Umbilia tomdarraghi sp. nov.

Taxon classificationAnimaliaLittorinimorphaCypraeidae

B69D63D6-7CEB-5C0C-B006-DBFFE6CFFF02

https://zoobank.org/45ADDEA9-CBF4-4FB7-A3FB-E1E34ED0EEF8

**Figure 1.**
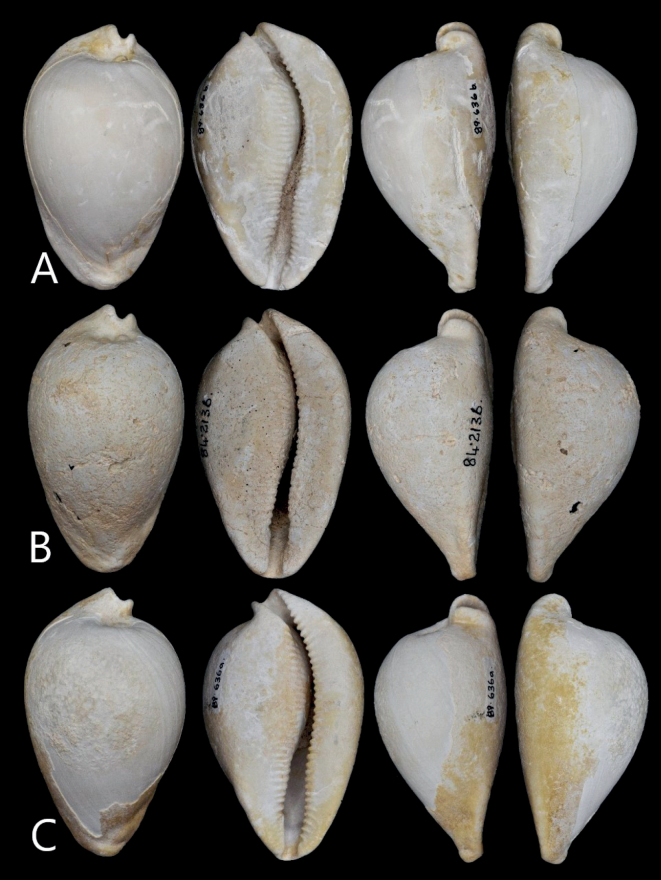
*Umbiliatomdarraghi* sp. nov.; dorsal, ventral and marginal aspects **A** holotype WAM 89.636b **B** paratype 4, WAM 84.2136 **C** paratype 1, WAM 89.636a.

**Figure 2.**
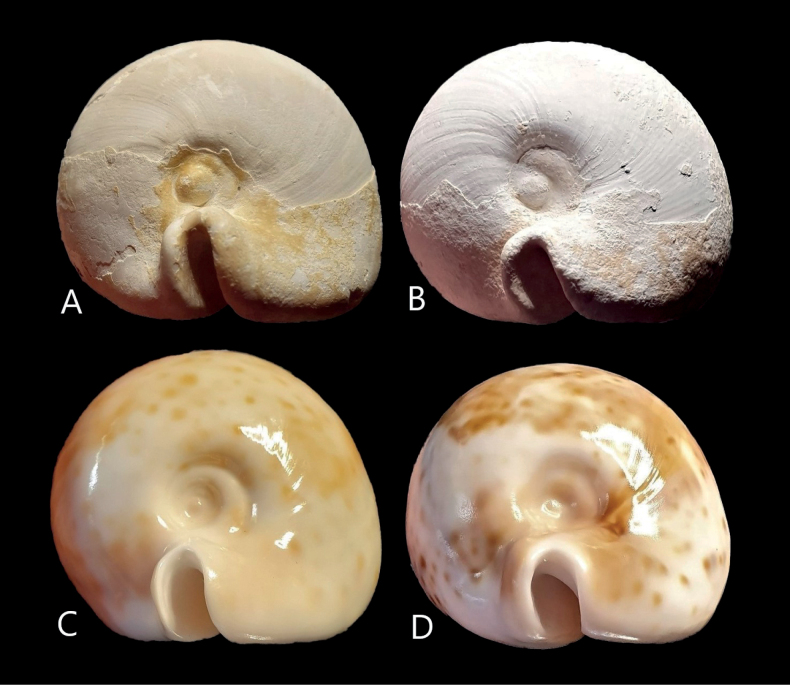
**A, B** detail of the spire and protoconch of *Umbiliatomdarraghi* sp. nov. **A** holotype WAM 89.636b **B** paratype 1, WAM 89.636a **C***U.hesitatahesitata***D***U.hesitatasuprastrata*.

Figs 1 , 2 , Table 1

Umbilia hesitata —Darragh 2002: 380, fig. 9 a–f.
Umbilia hesitata —Wilson and Clarkson 2004: 342, pl. 352, fig. b.
Umbilia hesitata —Goudey 2015: 41, figs b, c.
Umbilia hesitata —Lorenz 2018: 106, fig. 19.

###### Material examined.

***Holotype*.** Australia • Madura district, Roe Plains, Western Australia; October 1988; G.W. Kendrick leg.; dry specimen (fossil); among spoil material excavated from quarry, 2.5 km north of Hampton microwave repeater tower (31°56'34"S, 127°34'47"E); WAM 89.636b.

***Paratypes*.** Australia • 1; same location as holotype; October 1988; G.W. Kendrick leg.; dry specimen (fossil); WAM 89.636a (paratype 1) • 1; same location as holotype; October 1988; G.W. Kendrick leg.; dry specimen (fossil); WAM 89.636c (paratype 2) • 1; same location as holotype; October 1988; G.W. Kendrick leg.; dry specimen (fossil); MV P121294 (paratype 5) • 1; among spoil material in Main Roads quarry 16 km south of Madura Roadhouse, Madura (32°02'22"S, 127°02'50"E), Roe Plains, Western Australia; August 1985; G.W. Kendrick leg.; dry specimen (fossil); WAM 85.1462 (paratype 3) • 1; among spoil material in pit, 1.5 km north of Hampton microwave repeater tower (31°56'34"S, 127°34'47"E); October 1984; A. Rowe leg.; WAM 84.2136 (paratype 4) • 1; among spoil from foundation holes for Hampton microwave repeater tower (31°56'34"S, 127°34'47"E); April 1969; T.A. Darragh leg.; MV P302721 (paratype 6) • 1; same locality as preceding; June, 2004; P. Hunt leg.; PH collection (paratype 7) • 1; among spoil material alongside Eyre Highway, east of Madura, Western Australia, March 1995 (material probably sourced from Main Roads quarry 16 km south of Madura Roadhouse, Madura (32°02'22"S, 127°02'50"E); A. Bishop leg.; AB collection (paratype 8) • 1; among spoil material north of Hampton microwave repeater tower (31°56'26"S, 127°35'26"E); July 2007; C. Goudey leg.; CG collection (paratype 9) • 1; among spoil material at Hampton microwave repeater tower (31°57'52"S, 127°34'50"E); J. Fell leg.; JF collection (paratype 10).

###### Other material.

Australia; Roe Plains, same location as holotype; dry specimen (fossil); among spoil material; CG (1 repaired specimen).

###### Diagnosis.

Shell pyriform to ovately pyriform, humped; dorsal summit towards posterior, W/L = 59%, H/L = 48%; spire impressed; protoconch projecting and prominent, positioned to the left side and visible when the shell is viewed from a dorsal aspect. Coarse columellar teeth extending onto base. Anterior and posterior terminals extended; anterior lateral flanges well-developed, thickened; anterior extremity broad, flattened, rounded; anterior tips blunt. Anterior dorsal tubercules absent; a small, raised callus on left side only; anterior groove absent.

###### Description.

Of average shell length for the genus (76–87 mm; Table 1); shell pyriform to ovately pyriform, humped, with highest point towards posterior; W/L = 59.4%, H/L = 48.3%, and H/W = 81.3%. Shell formula [82(59-48-81) 20:17]. Anterior and posterior extremities extended; anterior extremity rounded, supported by broad, thickened, lateral flanges; anterior tips rounded, not pointed; posterior terminal curved to left with right posterior tip extending further; dorsum smooth. Single anterior tubercule evident as small, raised callus on left side only; anterior groove absent; base convex, broad, flattened anteriorly. Aperture widening anteriorly, narrowest at anterior end of posterior canal; apertural teeth coarse, evenly spaced, and well developed along whole length and on both sides of the aperture; columellar teeth extending onto base. Labral teeth (28–35) more numerous than columellar teeth (22–30). Shell margins rounded, smooth; spire umbilicate; protoconch large (4.8 ± 0.4 mm diameter; *n* = 6), projecting and prominent, positioned to the left so that the penultimate body whorl has greater exposure on the right side of the spire (Fig. 2A, B). Fossula narrow, smooth, and slightly concave.

**Table 1.** Descriptions and repositories of the type series of *Umbiliatomdarraghi* sp. nov.

**Specimens (repository)**	**Length (mm)**	**Width (mm)**	**Height (mm)**	**Columellar teeth**	**Labral teeth**
Holotype (WAM 89.636b)	86.8	52.1	41.7	30	35
Paratype 1 (WAM 89.636a)	83.5	49.8	40.4	28	33
Paratype 2 (WAM 89.636c)	83.6	50.2	40.6	25	31
Paratype 3 (WAM 85.1462)	76.6	45.5	36.6	22	30
Paratype 4 (WAM 84.2136)	87.3	50.7	40.6	26	31
Paratype 5 (MV P121294)	80.7	46.7	38.1	26	31
Paratype 6 (MV P302721)	80.8	47.2	39.1	26	33
Paratype 7 (PH)	85.5	52.2	41.2	28	32
Paratype 8 (AB)	80.6	48.3	39.5	26	28
Paratype 9 (CG)	78.0	47.0	39.0	23	29
Paratype 10 (JF)	79.0	46.2	38.5	26	32
Mean (± SD)	82.0 (± 3.6)	48.7 (± 2.4)	39.6 (± 1.5)	26.0 (± 2.2)	31.4 (± 2.0)

###### Differential diagnosis.

**Figure 3.**
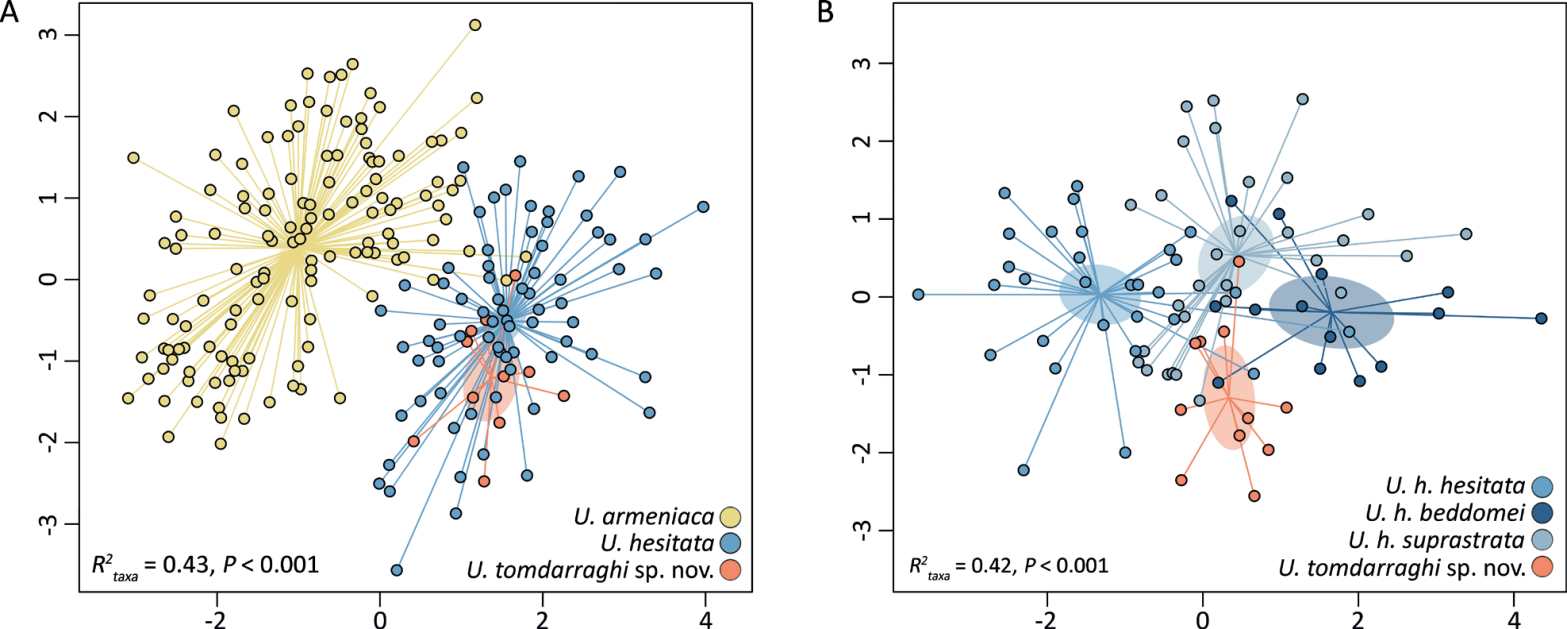
**A**nMDS ordination (stress = 0.14) of the resemblance matrix for *Umbiliaarmeniaca*, *U.hesitata* and *U.tomdarraghi* sp. nov. **B**nMDS ordination (stress = 0.19) of the resemblance matrix for the three *U.hesitata* subspecies and *U.tomdarraghi* sp. nov. Shaded ellipses indicate the 95% confidence interval of taxa (species or subspecies) centroids. The coefficient of determination (*R^2^*) and probability that distances between centroids arose by random chance (*P*) are presented.

**Figure 4.**
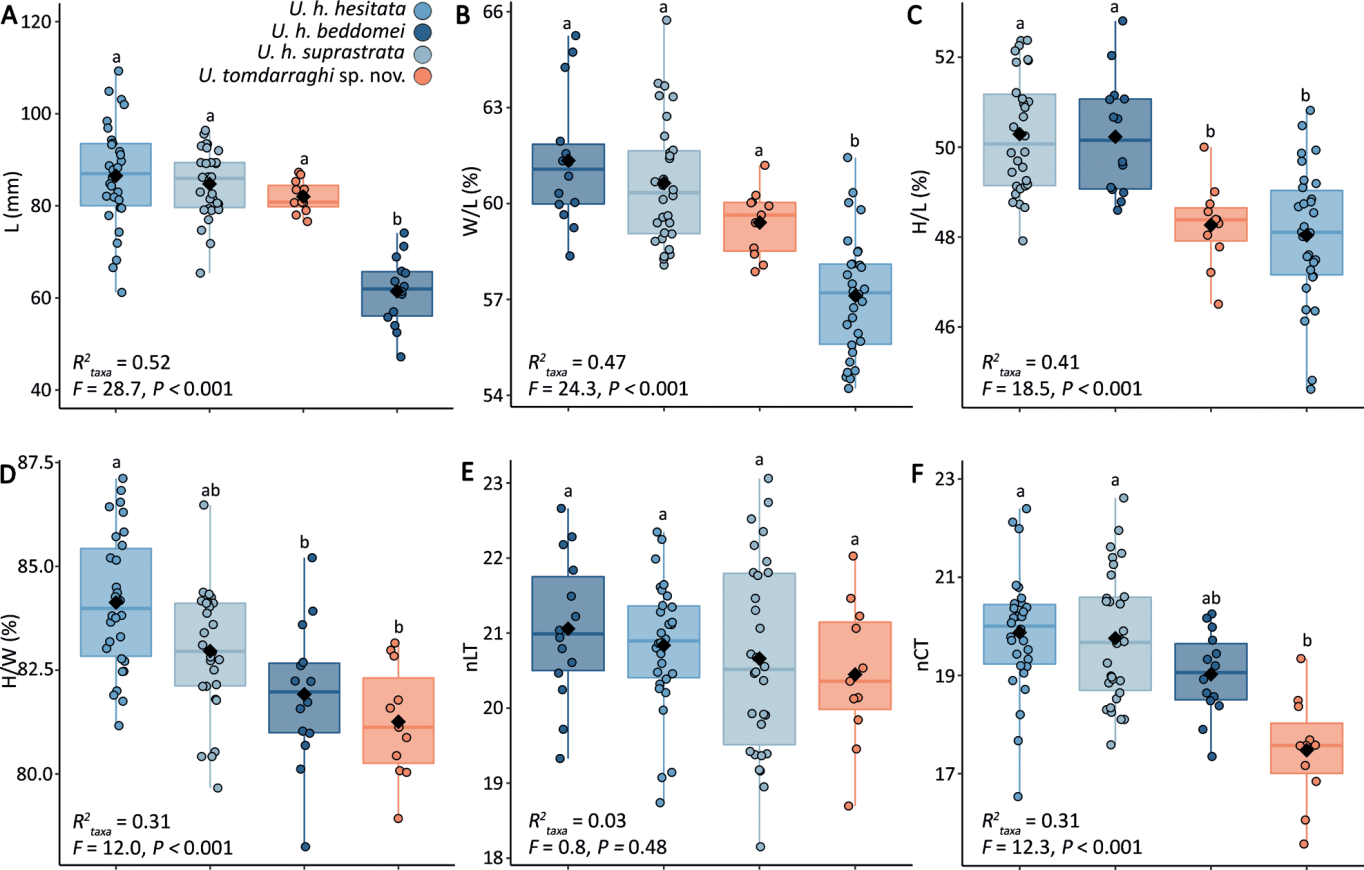
Box plots showing univariate comparisons of **A** length (L) **B** width to length ratio (W/L) **C** height to length ratio (H/L) **D** height to width ratio (H/W) **E** normalised labral tooth count (nLT) and **F** normalised columellar tooth count (nCT) among the accepted subspecies of *Umbiliahesitata* (*U.h.hesitata*, *U.h.beddomei*, *U.h.suprastrata*) and *U.tomdarraghi* sp. nov. Diamonds represent group means, boxes illustrate first and third quartile as box edges and median as central line. Shared superscripts identify means that are not statistically different (Holm-adjusted *P* ≥ 0.01) among taxa.

When compared to extant *Umbiliaarmeniaca* and *U.hesitata*, shell form of *U.tomdarraghi* sp. nov. is morphometrically more similar to *U.hesitata* (*F* = 7.9, *R*^2^ = 0.09, *P* < 0.001) than to *U.armeniaca* (*F* = 23.2, *R*^2^ = 0.14, *P* < 0.001, Fig. 3A). The explicit distinction between *U.tomdarraghi* sp. nov. and *U.hesitata*, when independently assessed using morphometric data for all three *U.hesitata* subspecies (*U.hesitatahesitata*, *U.h.beddomei* and *U.h.suprastrata*), showed clear separation (*F* = 6.9, *R*^2^ = 0.08, *P* < 0.001), with *U.hesitata* being significantly more variable in shell form (*F* = 10.1, *P* < 0.01) than *U.tomdarraghi* sp. nov. (Fig. 3B). When compared to individual subspecies of *U.hesitata*, shell form of *U.tomdarraghi* sp. nov. is most similar to *U.h.suprastrata*, but *U.h.suprastrata* is more similar to both *U.h.hesitata* and *U.h.beddomei* than it is to *U.tomdarraghi* sp. nov. (Fig. 3B; Table 2). It is notable that variability in shell form among the type series specimens of *U.tomdarraghi* sp. nov. is similar to that of *U.h.hesitata*, *U.h.beddomei*, and *U.h.suprastrata* (Table 3). Univariate comparisons of L, W/L, H/L, H/W, nLT, and nCT showed *U.tomdarraghi* sp. nov. to differ from *U.h.hesitata* by significantly greater W/L, lower H/W and lower nCT, from *U.h.beddomei* by significantly greater L and lower H/L, and from *U.h.suprastrata* by significantly lower H/L and lower nCT (Fig. 4). Key conchological features differentiating *U.tomdarraghi* sp. nov. from *U.h.hesitata*, *U.h.beddomei*, and *U.h.suprastrata* include coarser, extended columellar teeth; the broader, flatter, blunter anterior extremity; lack of both distinct anterior dorsal tubercules and an anterior groove; a flatter base; and a much larger, protruding protoconch (Table 4).

**Table 2.** PERMANOVA results testing the hypotheses that there were no differences in central tendency (i.e., centroid) of shell form among the *Umbiliahesitata* subspecies and *U.tomdarraghi* sp. nov. The Euclidean distance (*D*) between centroids, coefficient of determination (*R^2^*), and Holm-adjusted probability that the distance between centroids arose by random chance (*P*) are presented.

*Umbilia* sp./ssp.	*U.h.hesitata*			*U.h.beddomei*			*U.h.suprastrata*		
	*D*	*R^2^*	*P*	*D*	*R^2^*	*P*	*D*	*R^2^*	*P*
*U.h.beddomei*	3.21	0.36	0.001	–	–	–	–	–	–
*U.h.suprastrata*	2.03	0.19	0.001	2.08	0.19	0.001	–	–	–
*U.tomdarraghi* sp. nov.	2.52	0.25	0.001	2.51	0.35	0.001	2.27	0.21	0.001

**Table 3.** Permutation-based test results testing the hypotheses that there were no differences in variation (i.e., dispersion) in shell form among accepted *Umbiliahesitata* subspecies and *U.tomdarraghi* sp. nov. The mean (x̄) ± standard deviation (SD) and range in Euclidean distance that specimens were from their centroid are presented. Means with shared superscripts are not significantly (Holm-adjusted *P* ≥ 0.01) different.

*Umbilia* sp./ssp.	Distance from centroid*	
	(x̄ ± SD)	Range
*U.h.hesitata*	1.95 ± 0.68^a^	0.92–3.58
*U.h.beddomei*	1.77 ± 0.59^a^	0.84–3.05
*U.h.suprastrata*	2.01 ± 0.61^a^	0.86–3.40
*U.tomdarraghi* sp. nov.	1.43 ± 0.45^a^	0.67–2.15

**Table 4.** Comparison of key conchological features of *Umbiliahesitatahesitata*, *U.h.beddomei*, *U.h.suprastrata* and *U.tomdarraghi* sp. nov.

Feature:	*Umbiliahesitatahesitata*	*U.hesitatabeddomei*	*U.hesitatasuprastrata*	*U.tomdarraghi* sp. nov.
Columellar teeth:	Fine, restricted to aperture.	Fine, restricted to aperture.	Fine, restricted to aperture.	Coarse, extending onto base.
Anterior extremity:	Extended, rostrate, tapering; anterior tips somewhat pointed.	Shorter, broader; often callused.	Similar to *U.h.hesitata* but less extended.	Broader, flattened and rounded; anterior tips blunt.
Anterior dorsal tubercules:	Two tubercules separated by sulcus.	Two tubercules separated by sulcus.	Two tubercules separated by sulcus.	Indistinct left-side dorsal callus; no sulcus.
Posterior extremity:	Rostrate, pointed.	Shorter, less extended than *U.h.hesitata*.	Less extended than *U.h.hesitata*.	Less extended than *U.h.hesitata*.
Base:	Convex, flattened anteriorly.	Convex, flattened anteriorly.	More convex than *U.h.hesitata*; less flattened anteriorly.	Less convex and broader than *U.h.hesitata*; flattened anteriorly.
Aperture:	Widening slightly towards anterior.	Widening slightly towards anterior	Narrower than *U.h.hesitata*.	Narrower than *U.h.hesitata*; slightly constricted at anterior end of posterior canal.
Spire:	Spire impressed. Protoconch not protruding; positioned centrally (Fig. 2C).	Spire impressed. Protoconch not protruding; positioned centrally.	Spire impressed. Protoconch not protruding; positioned centrally (Fig. 2D).	Spire less impressed, broader. Protoconch much broader, protruding; positioned towards left side (Fig. 2A, B).
Anterior labral teeth:	Lengthening	Lengthening	Lengthening	Not lengthening
Shell formula:	87 [57-48-84] 21:20 (n=46)	61 [61-50-82] 21:19 (n=14)	85 [61-50-83] 21:20 (n=30)	82 [59-48-81] 20:17 (n=11)

A second species of *Umbilia*, *U.fodinata* (Darragh, 2011), occurs with *U.tomdarraghi* sp. nov. within the Roe Calcarenite. While Darragh (2011) originally assigned this species to the genus *Zoila* Jousseaume, 1884, in the most recent review of the family, Lorenz (2017, 2018) placed the species within *Umbilia* and this position is adopted here. Like *U.tomdarraghi* sp. nov., the spire of *U.fodinata* protrudes beyond the last shell whorl, but it is readily distinguished from *U.tomdarraghi* sp. nov. by its smaller size (72 mm), shell form (W/L 65%; H/L 54%), well-developed fossula, less extended posterior extremity, more tapered and shorter anterior extremity, and in the structure of the anterior-most columellar teeth, which are longer and coarser than those elsewhere on the columella and extend further onto the base (Darragh 2011).

Pliocene strata of the Cameron Inlet Formation at Flinders Island, off the north-east coast of Tasmania, around 2,000 km from the Roe Plains, contain at least three species of cowries, including two species of *Umbilia*: *U.furneauxensis*Southgate et al., 2021 and *U.hesitata* (Sutherland and Kershaw 1970; Darragh 1985; Southgate et al. 2021; Southgate and Roberts 2022). *Umbiliafurneauxensis* differs from *U.tomdarraghi* sp. nov. by its much smaller size (<60 mm), extension of apertural dentition to at least midway on the base and labrum, and heavily callused margins that may form a dimpled surface extending to the base. Flinders Island fossils assigned to *U.hesitata* can be distinguished from *U.tomdarraghi* sp. nov. by their much greater extension of both anterior and posterior extremities, restriction of their finer columellar teeth to the aperture, lengthening of the anterior-most labral teeth, a more convex base, and a more umbilicate spire with less prominent protoconch. However, comparison of *U.tomdarraghi* sp. nov. with *U.hesitata* from the Cameron Inlet Formation is done with caution at this stage because available specimens (*n* = 16, MV collection) vary considerably in shell form and conchological features to such an extent that they may not represent a single taxon.

*Umbiliatomdarraghi* sp. nov. superficially resembles the Miocene species *U.eximia* (G.B. Sowerby I, 1845) and *U.hallani* Hawke, 2020. It differs from the former by its much less prominent extremities, lack of prominent anterior dorsal tubercules, and by differences in the structure of the columellar teeth which, in *U.eximia*, are generally broad, deeply incised, and rectangular in cross section. *Umbiliahallani* is readily distinguished from *U.tomdarraghi* sp. nov. by its much smaller size with a more inflated body whorl and rostrate anterior extremity, and by the moderately formed anterior dorsal tubercules, separated by a diagonal groove.

###### Etymology.

Named to honour Dr T.A. Darragh, invertebrate paleontologist at Museums Victoria, Melbourne, Australia, in recognition of his significant contribution to our understanding of Australian marine molluscs, both fossil and living.

###### Distribution.

Known only from the Roe Calcarenite of the Roe Plains, Western Australia.

### ﻿Key to Pliocene *Umbilia* species

The four known Pliocene *Umbilia* species are described in the following key.

**Table:** 

1	Columellar teeth restricted to aperture margin	**4**
–	Columellar teeth extending somewhat onto base	**2**
2	Dorsal summit central; spire slightly umbilicate to flat; dentition extending as ridges to at least midway on the base; calloused margins with indentations; <60 mm	***U.furneauxensis***
–	Dorsal summit towards posterior	**3**
3	Globose, spire protruding beyond last whorl; posterior canal short, anterior canal very short, truncated; anterior-most columellar teeth longer and coarser than other columella teeth; well-developed, concave fossula	***U.fodinata***
–	Spire impressed, protoconch projecting and prominent; anterior terminal broad, flattened, rounded; anterior tips blunt; columellar teeth extending onto base; fossula narrow, concave	***U.tomdarraghi* sp. nov.**
4	Extremities rostrate; columella teeth fine, restricted to aperture; labral teeth lengthening anteriorly; traces of two anterior dorsal tubercules separated by sulcus; spire umbilicate, protoconch not protruding; fossula very narrow	***U.hesitata***

## ﻿Discussion

The genus *Umbilia* is well represented in the fossil record with at least 11 recognised species. Of the five extant *Umbilia* species (Lorenz 2017), *U.hesitata* is the only one with a fossil record (Darragh 2002; Goudey 2015). Darragh (2002) noted that although Late Miocene and Early Pliocene specimens of *U.hesitata* are uncommon, and often fragmentary or poorly preserved, sufficient well-preserved material is available to confirm their identification. Although similar to *U.hesitata* and previously assigned to that species (Darragh 2002; Wilson and Clarkson 2004; Goudey 2015; Lorenz 2018), *U.tomdarraghi* sp. nov. is morphometrically distinct from *U.hesitata* and differs in shell form and key conchological features, some of which (e.g., coarser teeth and flatter base) have been noted in previous studies (Wilson and Clarkson 2004; Lorenz 2017). As outlined above, there has been speculation in prior studies regarding the relationship between *U.tomdarraghi* sp. nov., living populations of *U.hesitata* found to the east, and living *U.armeniaca* found in adjacent waters in southern Western Australia. Our results clarify that *U.tomdarraghi* sp. nov. has greater affinity with *U.hesitata* than with *U.armeniaca*. Of the *U.hesitata* subspecies, our results also show closest affinity between *U.tomdarraghi* sp. nov. and the western most subspecies of *U.hesitata*, *U.h.suprastrata*, which has the closest natural range to the Roe Plains.

Possible lineages within the *Umbilia* have been a source of speculation in a number of studies (e.g., Darragh 2002; Wilson and Clarkson 2004; Yates 2008; Hawke 2020). Darragh (2002) noted that *U.hesitata* probably descended from *U.eximia*, and Wilson and Clarkson (2004) reasoned that there was progressive change from the ancestral *U.eximia* towards contemporary *U.hesitata*, where intermediate stages within this lineage were represented by separate species. In considering the likely position of *U.tomdarraghi* sp. nov. within this lineage, the possibility of a close ancestral relationship between *U.tomdarraghi* sp. nov. and living *U.hesitata* was considered a likely scenario at the start of this study. However, accepting the existence of *U.hesitata* within both the Miocene and Pliocene (Darragh 2002), and considering the clear morphometric separation of *U.tomdarraghi* sp. nov. and extant *U.hesitata* shown in this study, we consider it more likely that *U.tomdarraghi* sp. nov. is an offshoot from the *U.eximia*–*U.hesitata* lineage that became extinct in the Pliocene. Our results suggest that *U.hesitata* may not be present among the fauna of the Roe Calcarenite, greatly reducing the previously accepted distribution of this species within the fossil record.

## Supplementary Material

XML Treatment for Umbilia

XML Treatment for Umbilia tomdarraghi

## ﻿Additional information

### Author ORCIDs

Paul C. Southgate https://orcid.org/0000-0002-3781-2606

Thane A. Militz https://orcid.org/0000-0002-6476-8559

### Data availability

All of the data that support the findings of this study are available in the main text or Supplementary Information.
